# The Hungarian DREEM: translation, cultural adaptation, and psychometric validation of the learning environment questionnaire for medical and health professions education

**DOI:** 10.3389/fmed.2026.1788784

**Published:** 2026-06-17

**Authors:** Anna Dávidovics, Gergely Csaba, Nelli Farkas, Zsuzsanna Varga, Zsuzsanna Füzesi, Timea Németh

**Affiliations:** 1 Department of Languages for Biomedical Purposes and Communication, University of Pécs Medical School, Pécs, Hungary; 2 Department of Behavioural Sciences, University of Pécs Medical School, Pécs, Hungary; 3 Institute of Bioanalysis, University of Pécs Medical School, Pécs, Hungary

**Keywords:** cross-cultural validation, DREEM, health professions education, Hungary, learning environment, medical education

## Abstract

**Introduction:**

The learning environment is a critical component of health professions education, influencing student well-being, academic performance, and the long-term quality of the healthcare workforce. Reliable and culturally appropriate instruments are essential for monitoring educational environments and informing institutional and system-level quality improvement. Although the Dundee Ready Education Environment Measure (DREEM) has been widely used internationally, a validated Hungarian version has not previously been available.

**Methods:**

The DREEM questionnaire was translated and culturally adapted into Hungarian following established international guidelines for cross-cultural instrument validation. An online survey was administered to medical, dental, and pharmacy students at the University of Pécs Medical School between November and December 2024. Psychometric evaluation included assessment of internal consistency (Cronbach’s α), test-retest reliability (intraclass correlation coefficient), and confirmatory factor analysis to examine construct validity.

**Results:**

A total of 529 valid responses were included in the analysis. The Hungarian DREEM demonstrated excellent overall internal consistency (Cronbach’s α = 0.95) and good to excellent temporal stability (ICC range: 0.77–0.91). Confirmatory factor analysis provided only partial support for the original five-factor structure, with acceptable absolute fit indices but suboptimal incremental fit indices. The overall mean DREEM score (*M* = 135.0) indicated a generally positive perception of the learning environment.

**Conclusion:**

The Hungarian version of the DREEM demonstrated strong reliability and acceptable practical utility for assessing students’ perceptions of the learning environment in medical and health professions education. Its application can support institutional evaluation, national benchmarking, and international comparison, contributing to evidence-based educational quality assurance and health workforce development in Hungary. However, confirmatory factor analysis provided only partial support for the original five-factor structure, and construct validity should therefore be interpreted cautiously.

## Introduction

The educational environment plays a critical role in shaping the quality of higher education, including medical and health professions education programmes. Its impact on the attitudes, knowledge, and skills of medical and health professions students is profound, making its evaluation essential for designing and delivering high-quality curricula (1). As Isba et al. (2) noted, a supportive educational environment leads to improved learning outcomes. Consequently, assessing the educational environment is paramount to the success of higher education institutions.

Among the various instruments developed for this purpose, the Dundee Ready Education Environment Measure (DREEM) inventory has emerged as one of the most widely used and validated tools. Developed by Roff et al. (3), the DREEM inventory offers a universal method for assessing students’ perceptions of their educational environment, irrespective of cultural or institutional differences. It consists of 50 questions divided into five key domains: perceptions of learning, perceptions of teachers, academic self-perception, perceptions of the atmosphere, and social self-perception. Other than medical education (4–11), this tool has been successfully employed across various healthcare disciplines, including, but not limited to dentistry, optometry, occupational therapy, and physiotherapy (12–16).

The inventory’s global utility and adaptability are underscored by its translation and validation in numerous languages (17), including German (18), Greek (19), Korean (20), Polish (13) and Swedish (21).

However, the effective use of the DREEM inventory in different languages and cultural contexts requires careful translation, cultural adaptation, and psychometric validation. These processes are essential to preserve the original meaning of the instrument while ensuring its content validity and conceptual integrity across diverse cultures.

In Hungary, there has been a growing emphasis on monitoring and improving the quality of medical and health professions education, with the evaluation of the educational environment becoming one of the key components in this effort.

Despite the widespread use of the DREEM inventory globally, no validated Hungarian version currently exists, and only the official English version has been used (22–24). This reliance on English versions represents a significant gap in the assessment tools available for Hungarian institutions. Despite the broad international validation of the DREEM, a culturally adapted Hungarian version has been missing, limiting the ability of educators and researchers in Hungary to evaluate and benchmark the learning environment. As Alfakhry et al. (25) emphasize, using questionnaires in students’ native languages can make them more accessible, even when students are proficient in English. Recent studies have also highlighted the importance of evaluating the educational environment from the students’ perspective to improve learning outcomes (26, 27). Therefore, developing a validated Hungarian version of the DREEM inventory would enable educators, researchers, and policymakers to better assess and improve the experiences of medical and health professions students in Hungary.

This study seeks to address the absence of a validated Hungarian version of the DREEM by producing a culturally and linguistically adapted instrument tailored to the Hungarian medical education context. The primary objective was to translate, adapt, and psychometrically validate the tool, ensuring its reliability, structural validity, and conceptual equivalence for assessing students’ perceptions of their educational environment.

## Materials and methods

This study utilized a cross-sectional design to validate the DREEM questionnaire in Hungarian.

The Dundee Ready Education Environment Measure (DREEM) consists of 50 items designed to assess students’ perceptions of the educational environment across five domains: Students’ Perceptions of Learning, Students’ Perceptions of Teachers, Students’ Academic Self-Perceptions, Students’ Perceptions of Atmosphere, and Students’ Social Self-Perceptions. Each item is rated on a 5-point Likert scale ranging from 0 (strongly disagree) to 4 (strongly agree). Higher scores indicate more positive perceptions of the educational environment. Nine items in the instrument are negatively worded and require reverse scoring to ensure that higher scores consistently represent more favorable perceptions. These items are therefore recoded prior to analysis (4→0, 3→1, 2→2, 1→3, 0→4). The total DREEM score ranges from 0 to 200, with higher scores indicating a more positive overall educational environment. An example item from the questionnaire is: “The teaching is student-centered.” The full questionnaire is provided in the Supplementary Materials.

The domain scores were calculated by summing the corresponding items within each subscale according to the original scoring guidelines. Following established guidelines for the cross-cultural adaptation and validation of self-reported instrument (28–30), careful attention was given to the fact that a questionnaire cannot be translated literally. Instead, the primary challenge was to adapt it in a culturally appropriate and comprehensible manner while preserving the original meaning and intent of its items (31).

The methodology for the translation and psychometric validation of the Hungarian DREEM was informed by internationally accepted guidelines for cross-cultural adaptation of self-report instruments (29) and was aligned with approaches used in previous national adaptations of the DREEM questionnaire, including Greek (19), German (18), Swedish (21), and Polish (13).

The Hungarian validation of the DREEM questionnaire was a multifaceted process comprising several key steps: obtaining permissions, conducting forward and back translations, performing cultural adaptation, pre- and pilot testing, cognitive interviewing and undertaking psychometric analyses to assess its reliability, validity, and overall suitability for evaluating the educational environment in the Hungarian medical and health professions context.

### Cross-cultural adaptation

To validate the instrument in Hungarian, the initial step involved securing permission from the authors of the original DREEM questionnaire (3). Subsequently, ethical approval was obtained from the Regional Research Ethics Committee (reference number: 9996 - PTE 2024).

The DREEM questionnaire was translated into Hungarian following a standardized forward-backward translation methodology (28–31). Two bilingual translators, fluent in Hungarian and English and familiar with medical and health professions education terminology, independently translated the questionnaire into Hungarian. The translations were then synthesized into a single forward translation by a review committee comprising experts in medical education, health professions education, and linguistics. Back-translation into English was performed by two different bilingual translators who were blinded to the original questionnaire and had not participated in the forward translation process. Discrepancies between the original English version and the back-translated version were reviewed and resolved through consensus-based discussion by the expert committee to ensure semantic, conceptual, and cultural equivalence. Particular emphasis was placed on refining item wording to achieve the greatest possible clarity and reduce the potential for misinterpretation. A pre-test of the Hungarian version of the DREEM questionnaire was conducted with a convenience sample of 32 medical, dental, and pharmacy students. Participants completed the questionnaire and subsequently participated in cognitive debriefing interviews focusing on item clarity, comprehensibility, interpretability, and cultural appropriateness. Students were encouraged to identify ambiguous wording or expressions that were difficult to understand in Hungarian. Minor adjustments were made based on their feedback.

### Data collection

The finalized Hungarian version of the DREEM questionnaire was converted into a Google Form survey and distributed online to medical, dental, and pharmacy students across all years of study, ensuring comprehensive representation of various stages of medical education. Data collection was carried out over 1 month, between November 10 and December 10, 2024. To assess the temporal stability of the instrument, a subsample of students completed the DREEM questionnaire a second time, with a 2-week interval between administrations.

### Ethical considerations

This study involved human participants and received ethical approval from the Regional Research Ethics Committee (reference number: 9996 - PTE 2024). Information about the aim of the research was shared with all participants. Participation was voluntary, informed consent was obtained by ensuring all participants of anonymity, confidentiality, and data protection. Participants could withdraw at any time without consequences. The study complied with the Declaration of Helsinki.

### Psychometric evaluation

To assess the construct validity of the Hungarian version of the DREEM questionnaire, a confirmatory factor analysis (CFA) was conducted to examine the original five-factor structure proposed by Roff et al. (3) using robust maximum likelihood estimation. Given that previous cross-cultural adaptations of the DREEM have reported inconsistent structural findings, an exploratory factor analysis (EFA) was also conducted as a complementary exploratory approach. The five latent factors included: Students’ Perceptions of Learning (SPL), Students’ Perceptions of Teachers (SPT), Students’ Academic Self-Perceptions (SASP), Students’ Perceptions of Atmosphere (SPA), and Students’ Social Self-Perceptions (SSSP).

Model fit was assessed using multiple indices: the Comparative Fit Index (CFI), Tucker-Lewis Index (TLI), Root Mean Square Error of Approximation (RMSEA), and Standardized Root Mean Square Residual (SRMR). Acceptable thresholds were based on conventional guidelines (32): CFI and TLI ≥0.90, RMSEA ≤0.08, and SRMR ≤0.08.

Additional structural models were tested, including a higher-order CFA and a bifactor model. EFA was conducted using maximum likelihood extraction with oblimin rotation, with factor retention based on parallel analysis and scree plot inspection. An EFA-informed CFA model was subsequently specified based on primary loadings.

Internal consistency was evaluated using Cronbach’s alpha coefficients for the total scale and for each subscale. A threshold of α≥0.70 was used to indicate acceptable reliability (33), although slightly lower values were tolerated in the case of short subscales.

To examine the stability of the instrument over time, test-retest reliability was assessed using intraclass correlation coefficients (ICCs) based on a two-way mixed-effects model with absolute agreement. A subsample of students completed the Hungarian DREEM questionnaire twice with a 2-week interval between administrations. ICC values were interpreted following (34), with values below 0.5 indicating poor reliability, 0.5–0.75 moderate, 0.75–0.9 good, and values above 0.9 excellent reliability.

### Data analysis

All statistical analyses were conducted using R software (version 4.5.0). Descriptive statistics were calculated for demographic variables, each survey item and subscale, including means, standard deviations, and item-total correlations. Only fully completed questionnaires were included in the analyses, therefore, no imputation procedures were required for missing data. Items with reversed wording were recoded before analysis, in accordance with the original DREEM scoring guidelines. CFA was performed using the *lavaan* package in R (35). Model parameters were estimated using the robust maximum likelihood (MLR) estimator, appropriate for ordinal Likert-scale data. Exploratory factor analysis and parallel analysis were conducted using the psych package. ICCs were computed for each subscale using the *irr* packages in R (36), providing estimates of the instrument’s temporal stability.

## Results

### Sample characteristics

A total of 529 students completed the Hungarian version of the DREEM questionnaire. The majority of the respondents were female (70%), with 29% identifying as male, and 0.4% selecting another or undisclosed gender category. Most participants (76%) were aged between 18 and 21 years, followed by 22–25 years (20%), 26 years or older (3.6%). Participants represented three academic programmes: 72% were enrolled in medicine, 14% in dentistry, and 14% in pharmacy. Thus, the validation sample primarily consisted of medical students, while dental and pharmacy students represented smaller but comparable subgroups. Students from all years of study were represented, with the highest proportions from the 1st (56%) and 2nd (22%) years, followed by 3rd year (9.8%), 4th year (6.8%), and smaller numbers in the 5th (3.2%) and 6th (1.5%) years. The distribution of academic years differed among the three majors: dental and pharmacy students were predominantly in their first year, whereas among medical students, the majority were in the first and second years of study. Detailed demographic distributions by major are presented in Table 1.

**TABLE 1:** Characteristics of the study group by major (*n* = 529).

Characteristic	All majors	Medicine major	Dentistry major	Pharmacy major	*p*-value^a^
Number of participants	529	379	74	76	
Age - *n* (%)					0.732
18–21	402 (76.0%)	286 (75.5%)	56 (75.7%)	60 (79.0%)	
22–25	108 (20.4%)	79 (20.8%)	14 (18.9%)	15 (19.7%)	
26+	19 (3.6%)	14 (3.7%)	4 (5.4%)	1 (1.3%)	
Gender - *n* (%)					0.147
Male	155 (29.3%)	117 (30.9%)	14 (18.9%)	24 (31.6%)	
Female	372 (70.3%)	261 (68.9%)	60 (81.1%)	51 (67.1%)	
Undisclosed	2 (0.4%)	1 (0.3%)	0	1 (1.3%)	
Class year - *n* (%)^b^					<0.001
Year 1	296 (56.0%)	179 (47.2%)	55 (74.3%)	62 (81.6%)	
Year 2	136 (25.7%)	125 (33.0%)	9 (12.2%)	2 (2.6%)	
Year 3	41 (7.8%)	27 (7.1%)	9 (12.2%)	5 (6.6%)	
Year 4	31 (5.9%)	25 (6.6%)	0	6 (7.9%)	
Year 5	12 (2.3%)	10 (2.6%)	1 (1.4%)	1 (1.3%)	
Year 6	13 (2.5%)	13 (3.4%)	–	–	

^a^Pearson’s chi-squared test.

^b^The medicine major is a 6-year programme, while the dental and pharmacy majors are each 5-year programmes. As a result, Year 6 data are only applicable to the medicine major.

### Descriptive statistics

Descriptive statistics were calculated for each of the 50 DREEM items, as well as for the five subscales and the total score. Although descriptive DREEM scores are reported, these results are presented solely to characterize the validation sample and to allow comparison with previous DREEM validation studies. The present study was not designed to evaluate the educational environment of the institution, and the findings should not be interpreted as institutional benchmarking results. The total DREEM score in the full sample (*N* = 529) had a mean of 135.0 (SD = 26.8) out of a maximum possible score of 200. This falls within the “more positive than negative” range according to the DREEM interpretation guidelines (3). These results are comparable to those reported in previous DREEM validation studies and in the original DREEM study, in which the overall score was 132.4 (3). The subscale scores similarly reflect a generally favorable, although not excellent perception across the different dimensions of the learning environment (see Table 2). No floor or ceiling effects were observed: only 3.6% of students scored within the lowest or highest 10% of the total DREEM score range, scores ranged from 45 to 198, with a near-normal distribution.

**TABLE 2:** Descriptive statistics of the Hungarian DREEM validation sample (*n* = 529)*.

DREEM scores	Total score	M (SD)	Min-max	Interpretation
Global scale	200	135.0 (26.8)	45–198	More positive than negative
Subscales				
Perceptions of learning	48	29.9 (8.2)	0–48	A more positive perception
Perceptions of teachers	44	31.4 (6.1)	9–44	Moving in the right direction
Academic self-perceptions	32	21.0 (5.0)	6–32	Feeling more on the positive side
Perceptions of atmosphere	48	34.3 (6.8)	12–48	A more positive atmosphere
Social self-perceptions	28	18.3 (4.3)	4–28	Not too bad

M (SD), mean value (standard deviation); Min–max, minimal and maximal scores achieved. *Scores are presented for the translated and culturally adapted Hungarian DREEM used in the present validation study.

Items with the highest mean scores included Item 2 (“The teachers are knowledgeable” *M* = 3.56), Item 15 (“I have good friends in this school” *M* = 3.39), and Item 35 (“I find the experience disappointing” – reverse-scored, *M* = 3.39). Conversely, the lowest scoring items were Item 25 (“The teaching over-emphasizes factual learning” – reverse-scored, *M* = 1.46), Item 4 (“I am too tired to enjoy the course” – reverse-scored, *M* = 1.75), and Item 27 (“I am able to memorize all I need” *M* = 1.96). For detailed item-level statistics see Supplementary Table 1. These item-level results illustrate the distribution of responses within the validation sample and are presented to facilitate comparison with previous DREEM validation studies.

### Internal consistency

Internal consistency was assessed using both Cronbach’s alpha (α) and McDonald’s omega (ω) coefficients for each subscale and for the total DREEM scale (Table 3). The total DREEM scale demonstrated excellent reliability (α = 0.95; ω = 0.96). Across the five subscales, reliability estimates were consistently acceptable to good, with omega coefficients ranging from 0.75 to 0.90, supporting the internal consistency of the instrument. Four subscales met or exceeded the commonly accepted threshold of 0.70 for both α and ω (33). The Students’ Social Self-Perceptions (SSSP) subscale showed slightly lower reliability (α = 0.69; ω = 0.75), which remains acceptable, particularly given the relatively small number of items in this domain. Overall, the omega coefficients were comparable to or slightly higher than the corresponding alpha values, indicating robust reliability.

**TABLE 3:** Cronbach’s alpha and McDonald’s omega coefficients and intraclass correlation coefficients (ICCs) for the global scale and subscales in the Dundee Ready Education Environment Measure (DREEM).

DREEM	Number of items	Cronbach’s alpha (CI)	McDonald’s omega	ICC (CI)
Global scale	50	0.95 (0.94–0.96)	0.96	0.91 (0.87–0.93)
Subscales				
Perceptions of learning	12	0.88 (0.86–0.89)	0.90	0.88 (0.84–0.91)
Perceptions of teachers	11	0.82 (0.79–0.84)	0.86	0.84 (0.78–0.89)
Academic self-perceptions	8	0.73 (0.68–0.76)	0.77	0.77 (0.69–0.84)
Perceptions of atmosphere	12	0.83 (0.81–0.85)	0.88	0.81 (0.73–0.86)
Social self-perceptions	7	0.69 (0.65–0.73)	0.75	0.87 (0.82–0.91)

ICC, intraclass correlation coefficient; CI = 95% confidence interval.

### Test–retest reliability

To assess test–retest reliability, a subsample of 124 students completed the questionnaire a second time after a 2-week interval. The ICCs for the five subscales and the global scale indicated good to excellent reliability according to the commonly used interpretive benchmarks (Table 3) (34). The global scale demonstrated excellent test–retest reliability, with an ICC of 0.91. The Students’ Perceptions of Learning subscale demonstrated the highest stability (ICC = 0.88), followed closely by Students’ Social Self-Perceptions (ICC = 0.87). Other subscales also showed strong reliability: Students’ Perceptions of Teachers had an ICC of 0.84, Students’ Perceptions of Atmosphere had an ICC of 0.81, and Students’ Academic Self-Perceptions yielded an ICC of 0.77.

These results confirm the consistency of the DREEM scores over time and provide strong support for the reliability of the Hungarian version of the instrument across its five domains.

### Confirmatory factor analysis

The confirmatory factor analysis of the Hungarian DREEM provided partial support for the original five-factor structure. The chi-square statistic was significant, χ^2^(1117) = 4006.46, *p* < 0.001, indicating some deviation from perfect model fit - as is common with large sample sizes and complex models. The Comparative Fit Index (CFI) was 0.739 and the Tucker–Lewis Index (TLI) was 0.725, both falling below the conventional threshold of 0.90. However, other fit indices indicated more favorable results: the Root Mean Square Error of Approximation (RMSEA) was 0.070 with a 90% confidence interval of [0.068, 0.072] and *p* < 0.001, while the Standardized Root Mean Square Residual (SRMR) was 0.066. Additionally, the Goodness-of-Fit Index (GFI) and Adjusted GFI were high, at 0.946 and 0.939 respectively. Standardized factor loadings and factor correlations were extracted from the CFA model and are presented in Supplementary Tables 2, 3.

### Testing alternative models

A higher-order CFA model showed similar fit (CFI = 0.744, TLI = 0.731; RMSEA = 0.069, SRMR = 0.065), indicating no meaningful improvement over the original model.

The bifactor model did not converge and yielded inadmissible parameter estimates, suggesting that this structure was not supported in the present dataset.

Parallel analysis and inspection of the scree plot suggested a seven-factor solution. The EFA model showed good fit (TLI = 0.94, RMSEA = 0.031, RMSR = 0.02) and explained approximately 32% of the variance. However, the loading pattern was complex, with several cross-loadings and moderate factor correlations, indicating a multidimensional but not clearly separable structure. A CFA model based on the seven-factor solution showed improved, but still suboptimal, fit (CFI = 0.852, TLI = 0.843; RMSEA = 0.054, SRMR = 0.063). Inspection of item content revealed that several factors lacked conceptual coherence. For example, the first factor combined items from multiple original domains, reflecting heterogeneous constructs rather than a clearly interpretable dimension.

## Discussion

This study aimed to translate, culturally adapt, and psychometrically validate the Dundee Ready Education Environment Measure (DREEM) in Hungarian. The findings indicate that the Hungarian version demonstrates strong reliability and provides useful insights into students’ perceptions of their educational environment, while confirmatory factor analysis provided only partial support for the original five-factor structure. Although the instrument showed strong internal consistency and temporal stability, replication of the original latent structure was incomplete. As the first validated Hungarian adaptation of the DREEM, it fills an important gap in national medical and health professions education research and provides a standardized option for evaluating learning environments within Hungary. It may also support future cross-institutional and international comparisons using a contextually aligned instrument. The results also show broad similarities with several international validation studies, although such comparisons should be interpreted cautiously given differences in educational and cultural contexts (13, 20, 37–42).

The total score obtained in this study (M = 135.0, SD = 26.8) reflects an overall favorable perception of the learning environment and falls into the “more positive than negative” category. The descriptive score profile was broadly comparable to previous DREEM validation studies. As in other adaptations, the Social Self-Perceptions domain showed relatively lower scores, a pattern reported across multiple cultural contexts (37, 42).

The Hungarian version demonstrated excellent overall internal consistency (α = 0.95). Four out of five subscales exceeded the 0.70 threshold, while Social Self-Perceptions remained acceptable at α = 0.69. This profile is highly consistent with other adaptations, such as the Polish (13), Spanish (37), Vietnamese (42), Turkish (38), German (18), and Moroccan Arabic versions (40), all of which reported strong global reliability and some variability between subscales. Importantly, strong internal consistency and temporal stability indicate that the instrument measures constructs consistently over time, but these indices alone do not establish that the Hungarian DREEM reproduces the same latent structure as the original instrument. Reliability and construct validity therefore represent related but distinct psychometric properties. Accordingly, the strong reliability findings in the present study should not be interpreted as evidence of full structural replication of the original five-factor model. The test-retest analysis in this study produced ICC values between 0.77 and 0.91, indicating strong stability over time. This finding is consistent with the temporal stability reported in the Polish version (13). Although the Korean adaptation also supported the five-factor structure, it primarily highlighted issues with convergent and discriminant validity rather than test-retest reliability (20).

The CFA results indicated acceptable absolute model fit (RMSEA = 0.070; SRMR = 0.066), while incremental fit indices were below conventional thresholds (CFI = 0.739; TLI = 0.725), suggesting partial rather than full replication of the original five-factor structure. This pattern indicates that, although the model captures aspects of the data structure, its structural validity is limited. Several explanations may account for the modest incremental fit indices observed in the Hungarian sample. First, educational environment constructs may be interpreted differently across cultural and institutional contexts, particularly in relation to teacher-student relationships, perceptions of authority, and social support within medical education. Second, although the translation and adaptation process aimed to preserve conceptual equivalence, subtle linguistic differences may still influence how individual items are interpreted in Hungarian. Third, findings from previous cross-cultural validation studies suggest that some DREEM items may overlap conceptually or function differently across educational systems, contributing to instability in the original factor structure across cultures.

Comparisons with international validation studies should also be interpreted cautiously. Differences in educational systems, curricular organization, sample composition, translation procedures, and analytical approaches may substantially influence psychometric findings across adaptations. Consequently, similarities with previous international validation studies should be viewed as contextual and suggestive rather than confirmatory evidence of structural equivalence. Among cross-cultural validations, the Polish adaptation represents one of the strongest replications of the original model, demonstrating excellent fit (CFI = 0.964; TLI = 0.962; RMSEA = 0.043), indicating that full structural confirmation is achievable under certain conditions (13). However, most other studies have not reproduced such favorable model fit. Among studies employing CFA, the Korean validation is the most comparable to the present findings, reporting a similar pattern of acceptable absolute fit (RMSEA ≈ 0.060) but low incremental indices (CFI ≈ 0.74; TLI ≈ 0.73), with the model interpreted as only partially supported (20). The Moroccan Arabic adaptation further illustrates variability in confirmatory results across domains, suggesting that model fit may differ substantially depending on the subscale (40). Another European adaptation from Sweden reported poor CFA fit, reinforcing the difficulty of consistently reproducing the original structure using confirmatory methods (21).

In response to these challenges, a number of studies have proposed modified or shortened versions of the DREEM. Among those using CFA, the Malay adaptation concluded that the original five-factor structure was not a good fit and recommended a substantially shortened instrument (retaining 19 items), indicating considerable structural revision (39). Other studies have relied primarily on exploratory factor analysis (EFA) to derive alternative structures. The Turkish validation proposed a six-factor solution and removed a large number of items (retaining 23 items), resulting in a markedly reduced instrument (38). Similarly, the German (18) and Greek (19) adaptations identified factor structures that diverged from the original subscales, with item reallocations. The Swedish study initially attempted CFA but, due to poor model fit, proceeded with EFA and proposed an alternative five-factor solution with redistributed items (21).

To further investigate the dimensionality of the instrument, alternative structural models were explored. The higher-order CFA model did not meaningfully improve model fit relative to the original structure, suggesting that modeling the five domains under a broader higher-order construct did not provide a markedly superior representation of the data. The bifactor model yielded inadmissible parameter estimates and unstable solutions, indicating insufficient support for simultaneous modeling of a general factor and orthogonal domain-specific factors. Exploratory factor analysis suggested a more complex seven-factor structure with improved statistical fit, however, this solution was characterized by substantial cross-loadings and limited conceptual coherence. Several extracted factors included items from multiple theoretically distinct original domains, making the resulting factor structure difficult to interpret conceptually. A subsequent CFA based on the seven-factor solution showed improved, but still suboptimal, fit. Taken together, these findings suggest that although the underlying structure of the DREEM may be more complex than originally proposed, the alternative models did not provide a psychometrically robust and theoretically interpretable replacement for the original framework. These observations align with previous international validation studies indicating that full replication of the original DREEM factor structure is not consistently achieved across cultural contexts. Importantly, although several modified or alternative structures have been proposed, these have generally been developed within single-study contexts and have not yet been consistently replicated across independent samples. The original five-factor structure was retained with caution based on these and for several other reasons. First, the instrument was developed through a theory-informed process, and its five domains represent conceptually distinct dimensions of the educational environment. Second, modifications aimed at improving statistical fit - such as item removal or factor restructuring - may compromise content validity by narrowing the conceptual coverage of the instrument. Third, maintaining the original structure enables comparability with the extensive international DREEM literature.

The validated Hungarian DREEM provides a standardized tool for medical and health professions faculties to assess and improve their educational environments. Because the instrument produces both overall and domain-specific scores, it may help institutions identify areas where targeted improvements could be considered. For instance, subscale results may highlight aspects of the learning environment related to teaching practices, institutional atmosphere, or student support that warrant further attention. In addition, the Hungarian DREEM could be incorporated into institutional quality assurance and programme evaluation processes, for example through periodic administration during curriculum review cycles. Repeated use over time may also allow institutions to monitor whether curricular changes or educational reforms are associated with shifts in students’ perceptions of the learning environment. Comparable applications in Poland (13) and Romania (41) confirm that localized versions can reliably assess students’ perceptions of the educational environment. Such studies suggest potential for the use of localized DREEM versions in curriculum evaluation and national benchmarking. Importantly, using a culturally adapted instrument enhances accessibility and student engagement compared to reliance on the English version.

This study followed the framework of Beaton et al. (29) for cross-cultural adaptation, ensuring both linguistic and conceptual equivalence. Comparable methodological rigor is evident in the Korean adaptation, which also applied a multi-step process, and in the Polish study, which explicitly tested temporal stability (13, 20).

However, certain limitations must be acknowledged. Although the sample size was substantial (*N* = 529, ∼33% of the UPMS student population), it was not evenly distributed, as 56% of respondents were first-year students, which may have influenced overall results. Data collection was conducted online, meaning response behavior (e.g., completion time, engagement level) could not be controlled. Another limitation relates to the use of a self-administered questionnaire. As with other self-report instruments, responses to the DREEM may be influenced by social desirability bias, acquiescence tendencies, and contextual factors related to the timing and circumstances of data collection. Consequently, students’ reported perceptions may not fully reflect their underlying experiences, and these potential sources of response bias should be considered when interpreting the results. An important limitation of the present study relates to the confirmatory factor analysis results of the original and the alternative factor models. Within this broader international landscape, the Hungarian results align most closely with studies demonstrating acceptable absolute fit but modest incremental fit, supporting a cautious interpretation of structural validity while retaining the conceptual framework of the instrument. An additional limitation concerns the incomplete replication of the original five-factor structure. Although the instrument demonstrated strong reliability, the CFA findings suggest that the latent structure of the Hungarian DREEM may differ partially from the original conceptualization, and construct validity should therefore be interpreted cautiously.

### Future implications

While previous adaptations, such as the Korean and Vietnamese versions, have established strong reliability and identified problematic items, subgroup invariance testing has not yet been systematically undertaken (20, 42). Longitudinal applications may provide insights into how the Hungarian DREEM responds to curricular changes, while qualitative investigations could help clarify how students locally interpret constructs such as “atmosphere” or “self-perception.” Repeated administrations across cohorts or academic years could also allow institutions to monitor the educational impact of curricular reforms and identify emerging trends in students’ perceptions of the learning environment. Given the predominance of female participants in the present sample, future studies could also investigate measurement invariance across sex (e.g., configural and metric levels) to assess whether the Hungarian DREEM functions equivalently across subgroups and can therefore support reliable benchmarking and subgroup comparisons.

## Conclusion

The Hungarian version of the DREEM demonstrated strong internal consistency and temporal stability. However, confirmatory factor analysis provided only partial support for the original five-factor structure, indicating that evidence for construct validity remains limited. Although the instrument retained conceptual coherence and practical utility for evaluating students’ perceptions of the educational environment, the latent structure of the Hungarian DREEM should be interpreted cautiously. Further research is warranted to explore the underlying dimensional structure of the instrument and to determine whether culturally specific modifications or shortened versions may improve structural fit. Nevertheless, despite these limitations, the availability of a validated Hungarian version enables systematic evaluation of educational environments in medical and health professions education, allowing institutions to identify strengths and areas needing improvement. Its use could support evidence-based curriculum development, faculty training, and institutional quality assurance processes. Given its comprehensive, student-centered perspective, the DREEM may also serve as a valuable reference point for shaping educational strategy and informing curriculum designers, educational leaders, and quality assurance stakeholders. Refining selected items, extending its application to other health-related faculties, and longitudinally tracking students’ perceptions throughout their studies could further enhance its value as a practical tool for supporting continuous improvement in the quality of medical and health professions education in Hungary.

## Data Availability

The original contributions presented in this study are included in this article/Supplementary material, further inquiries can be directed to the corresponding author.
