# Supplementary material for: The Hungarian DREEM: translation, cultural adaptation, and psychometric validation of the learning environment questionnaire for medical and health professions education
Source: Front Med (Lausanne). 2026 Jun 17;13:1788784. doi: 10.3389/fmed.2026.1788784 (PMC13318560; doi:10.3389/fmed.2026.1788784)
Supplement: Supplementary file 1 [file Data_Sheet_1.docx]

**Supplementary material – DREEM questionnaire (Hungarian version)**

1. Nem

- Férfi
- Nő
- Nem szeretnék nyilatkozni

2. Életkor

- 18–21
- 22–25
- 26–29
- 30+

3. Szak

- általános orvos
- fogorvos
- gyógyszerész
- biotechnológus
- egyéb:

4. Évfolyam

- 1
- 2
- 3
- 4
- 5
- 6

DREEM kérdőív

Kérjük, jelölje meg, hogy az alábbi állításokkal mennyire ért egyet:

Egyáltalán nem értek egyet (1), Nem értek egyet (2), Bizonytalan vagyok (3), Egyetértek (4), Teljes mértékben egyetértek (5).

1. Sok bátorítást kapok, hogy aktívan részt vegyek az órákon.

(1) (2) (3) (4) (5)

2. Az oktatók nagy tudásúak.

(1) (2) (3) (4) (5)

3. A stresszel küzdő hallgatókat a kari támogató rendszer hatékonyan segíti.

(1) (2) (3) (4) (5)

4. Túl fáradt vagyok ahhoz, hogy élvezzem az órákat.

(1) (2) (3) (4) (5)

5. Azokat a tanulási módszereket, amiket korábban használtam, továbbra is jól tudom alkalmazni.

(1) (2) (3) (4) (5)

6. A klinikai oktatók türelmesek a betegekkel.

(1) (2) (3) (4) (5)

7. Az oktatás gyakran hat rám ösztönzően.

(1) (2) (3) (4) (5)

8. Az oktatók gúnyolódnak a hallgatókon.

(1) (2) (3) (4) (5)

9. Az oktatók tekintélyelvűek.

(1) (2) (3) (4) (5)

10. Biztos vagyok benne, hogy sikeresen el tudom végezni ezt az évet.

(1) (2) (3) (4) (5)

11. Nyugodt légkör jellemzi a klinikai gyakorlatokat.

(1) (2) (3) (4) (5)

12. Az egyetemen jól összeállított órarendünk van.

(1) (2) (3) (4) (5)

13. Az oktatás hallgatóközpontú.

(1) (2) (3) (4) (5)

14. Ritkán unatkozom az órákon.

(1) (2) (3) (4) (5)

15. Jó barátaim vannak ezen az egyetemen.

(1) (2) (3) (4) (5)

16. Az oktatás során kellő figyelmet fordítanak a kompetenciáim fejlesztésére.

(1) (2) (3) (4) (5)

17. A csalás komoly probléma ezen az egyetemen.

(1) (2) (3) (4) (5)

18. A klinikai oktatók megfelelően kommunikálnak a betegekkel.

(1) (2) (3) (4) (5)

19. A szociális életem jó.

(1) (2) (3) (4) (5)

20. Az oktatás lényegre törő.

(1) (2) (3) (4) (5)

21. Úgy érzem, hogy ezen az egyetemen jó szakmai felkészítést kapok.

(1) (2) (3) (4) (5)

22. Az oktatás megfelelően fejleszti az önbizalmam.

(1) (2) (3) (4) (5)

23. Nyugodt a légkör az előadások során.

(1) (2) (3) (4) (5)

24. Az oktatásra szánt időt hatékonyan használják ki.

(1) (2) (3) (4) (5)

25. Az oktatás során túl nagy hangsúlyt kap az elméleti anyag.

(1) (2) (3) (4) (5)

26. Az előző évben tanultak jól megalapozták ezt a tanévemet.

(1) (2) (3) (4) (5)

27. Képes vagyok mindent memorizálni, amire szükségem van.

(1) (2) (3) (4) (5)

28. Ritkán érzem magam magányosnak.

(1) (2) (3) (4) (5)

29. Az oktatók sok hasznos visszajelzést adnak a hallgatóknak.

(1) (2) (3) (4) (5)

30. Van lehetőségem az interperszonális készségeim fejlesztésére.

(1) (2) (3) (4) (5)

31. Sokat tanultam az empátia fontosságáról szakmai szempontból.

(1) (2) (3) (4) (5)

32. Az oktatók építő kritikát fogalmaznak meg ezen az egyetemen.

(1) (2) (3) (4) (5)

33. Az órákon jól érzem magam a többiek társaságában.

(1) (2) (3) (4) (5)

34. Nyugodt a hangulat a szemináriumok/gyakorlatok során.

(1) (2) (3) (4) (5)

35. Ez az egyetem csalódás számomra.

(1) (2) (3) (4) (5)

36. Jól tudok koncentrálni.

(1) (2) (3) (4) (5)

37. Az oktatók érthető példákat használnak.

(1) (2) (3) (4) (5)

38. Az órák céljai érthetőek számomra.

(1) (2) (3) (4) (5)

39. Az oktatók gyakran ingerültek az órákon.

(1) (2) (3) (4) (5)

40. Az oktatók jól felkészültek az órákra.

(1) (2) (3) (4) (5)

41. A problémamegoldó készségeimet hatékonyan fejlesztik ezen az egyetemen.

(1) (2) (3) (4) (5)

42. A pozitív élmények túlsúlyban vannak a tanulás okozta stresszel szemben.

(1) (2) (3) (4) (5)

43. A légkör motivál a tanulásban.

(1) (2) (3) (4) (5)

44. Az oktatók arra motiválnak, hogy aktív tanulói szerepet vállaljak.

(1) (2) (3) (4) (5)

45. Az elsajátítandó tananyagok többsége releváns a karrierem szempontjából.

(1) (2) (3) (4) (5)

46. Kényelmes a lakhelyem.

(1) (2) (3) (4) (5)

47. A hosszú távú tanulás nagyobb hangsúlyt kap, mint a rövid távú.

(1) (2) (3) (4) (5)

48. Az oktatás túlságosan oktató-központú.

(1) (2) (3) (4) (5)

49. Úgy érzem, hogy bátran feltehetem a kérdéseimet.

(1) (2) (3) (4) (5)

50. A hallgatók irritálják az oktatókat.

(1) (2) (3) (4) (5)

+1. Egyéb tényezők

Kérem, soroljon fel bármilyen egyéb tényezőt, ami Ön szerint hatással van az oktatási környezetre.
