# Supplementary material for: The Hungarian DREEM: translation, cultural adaptation, and psychometric validation of the learning environment questionnaire for medical and health professions education
Source: Front Med (Lausanne). 2026 Jun 17;13:1788784. doi: 10.3389/fmed.2026.1788784 (PMC13318560; doi:10.3389/fmed.2026.1788784)
Supplement: Supplementary file 2 [file Table_1.docx]

| **Subscale** | **Item** | **Medicine major (N=379)** | | **Dentistry major (N=74)** | | **Pharmacy major (N=76)** | |
| --- | --- | --- | --- | --- | --- | --- | --- |
|  |  | M | SD | M | SD | M | SD |
| SPL |  | 29.94 | 8.43 | 30.57 | 8.26 | 29.30 | 6.66 |
|  | 1. I am encouraged to participate in classes. | 2.67 | 1.04 | 2.82 | 1.08 | 2.64 | 1.01 |
|  | 7. The teaching is often stimulating. | 2.83 | 0.98 | 2.73 | 1.02 | 2.67 | 1.08 |
|  | 13. Teaching is student-centred. | 2.71 | 1.03 | 2.89 | 1.01 | 2.72 | 0.90 |
|  | 16. The teaching is sufficiently concerned to develop my competence. | 2.53 | 1.02 | 2.61 | 1.06 | 2.51 | 0.98 |
|  | 20. The teaching is well focused. | 2.50 | 1.12 | 2.68 | 1.05 | 2.51 | 0.98 |
|  | 21. The teaching is sufficiently concerned to develop my confidence. | 2.20 | 1.16 | 2.30 | 1.12 | 2.07 | 1.08 |
|  | 24. The teaching time is put to good use. | 2.64 | 1.07 | 2.89 | 0.86 | 2.75 | 0.93 |
|  | 25. The teaching overemphasises factual learning. | 1.46 | 1.06 | 1.41 | 1.01 | 1.50 | 0.95 |
|  | 38. I am clear about the learning objectives of the courses. | 2.80 | 0.95 | 2.95 | 0.91 | 2.68 | 0.85 |
|  | 44. The teaching encourages me to be an active learner. | 2.74 | 1.08 | 2.54 | 1.03 | 2.42 | 1.05 |
|  | 47. Long-term learning is emphasised over short-term. | 2.47 | 1.20 | 2.45 | 1.03 | 2.55 | 0.99 |
|  | 48. The teaching is too teacher-centred. | 2.41 | 1.01 | 2.31 | 1.05 | 2.26 | 0.98 |
| SPT |  | 31.50 | 6.04 | 31.55 | 6.45 | 31.07 | 5.68 |
|  | 2. The teachers are knowledgeable. | 3.58 | 0.63 | 3.54 | 0.70 | 3.47 | 0.77 |
|  | 6. The teachers are patient with patients. | 2.71 | 0.83 | 2.99 | 0.89 | 2.58 | 1.00 |
|  | 8. The teachers ridicule the students | 3.03 | 1.05 | 3.11 | 1.03 | 3.37 | 0.97 |
|  | 9. The teachers are authoritarian | 1.94 | 1.16 | 1.84 | 1.13 | 2.16 | 1.17 |
|  | 18. The teachers have good communications skills with patients. | 2.71 | 0.88 | 2.88 | 0.82 | 2.57 | 0.86 |
|  | 29. The teachers are good at providing feedback to students. | 2.44 | 1.08 | 2.42 | 1.00 | 2.47 | 0.95 |
|  | 32. The teachers provide constructive criticism here. | 2.64 | 0.96 | 2.49 | 0.96 | 2.45 | 0.86 |
|  | 37. The teachers give clear examples. | 3.01 | 0.82 | 2.88 | 0.80 | 2.87 | 0.80 |
|  | 39. The teachers get angry in class. | 3.19 | 0.90 | 3.11 | 0.99 | 3.22 | 1.06 |
|  | 40. The teachers are well prepared for their classes. | 3.33 | 0.77 | 3.27 | 0.81 | 3.20 | 0.78 |
|  | 49. The students irritate the teachers. | 2.92 | 0.96 | 3.04 | 1.06 | 2.71 | 1.18 |
| SASP |  | 21.09 | 4.94 | 21.20 | 4.76 | 20.26 | 5.09 |
|  | 5. Learning strategies which worked for me before continue to work for me now. | 2.64 | 1.02 | 2.58 | 1.08 | 2.59 | 1.00 |
|  | 10. I am confident about my passing this year. | 2.69 | 1.02 | 2.70 | 0.93 | 2.66 | 0.95 |
|  | 21. I feel I am being well prepared for my profession. | 3.26 | 0.82 | 3.45 | 0.84 | 3.18 | 0.74 |
|  | 26. Last year’s work has been a good preparation for this year’s work. | 2.60 | 1.03 | 2.57 | 0.96 | 2.30 | 1.16 |
|  | 27. I am able to memorise all I need. | 1.98 | 1.08 | 1.93 | 0.96 | 1.87 | 0.94 |
|  | 31. I have learned a lot about empathy in my profession. | 2.84 | 1.05 | 2.89 | 1.06 | 2.47 | 1.02 |
|  | 41. My problem solving skills are being well developed here. | 2.70 | 1.10 | 2.72 | 1.02 | 2.58 | 1.00 |
|  | 45. Much of what I have to learn seems relevant to a career in medicine. | 2.38 | 1.05 | 2.36 | 0.94 | 2.61 | 0.96 |
| SPA |  | 34.60 | 6.91 | 34.86 | 6.85 | 32.41 | 6.14 |
|  | 11. The atmosphere is relaxed during the ward teaching. | 2.54 | 0.83 | 2.77 | 0.91 | 2.50 | 0.91 |
|  | 12. This school is well timetabled. | 2.42 | 1.09 | 2.22 | 0.98 | 2.12 | 1.08 |
|  | 17. Cheating is a problem in this school. | 2.82 | 1.28 | 3.07 | 1.30 | 2.66 | 1.26 |
|  | 23. The atmosphere is relaxed during lectures. | 3.28 | 0.82 | 3.32 | 0.76 | 3.09 | 0.81 |
|  | 30. There are opportunities for me to develop interpersonal skills | 2.64 | 0.96 | 2.54 | 0.99 | 2.49 | 0.87 |
|  | 33. I feel comfortable in classes socially. | 3.30 | 0.87 | 3.28 | 0.67 | 3.07 | 0.95 |
|  | 34. The atmosphere is relaxed during seminars/tutorials. | 3.40 | 0.79 | 3.30 | 0.83 | 3.18 | 0.82 |
|  | 35. I find the learning experience disappointing. | 3.39 | 1.03 | 3.57 | 0.97 | 3.25 | 1.18 |
|  | 36. I am able to concentrate well | 2.44 | 0.97 | 2.51 | 0.79 | 2.33 | 0.94 |
|  | 42. The enjoyment outweighs the stress of studying medicine. | 2.50 | 1.15 | 2.46 | 1.04 | 2.24 | 1.07 |
|  | 43. The atmosphere motivates me as a learner. | 3.10 | 0.95 | 2.99 | 0.97 | 2.72 | 1.07 |
|  | 49. I feel able to ask the questions I want. | 2.78 | 1.03 | 2.84 | 1.04 | 2.76 | 0.92 |
| SSSP |  | 18.12 | 4.44 | 19.12 | 3.70 | 18.26 | 4.24 |
|  | 3. There is a good support system for students who get stressed. | 2.30 | 1.10 | 2.64 | 1.02 | 2.36 | 1.08 |
|  | 4. I am too tired to enjoy the courses. | 1.73 | 1.08 | 1.73 | 0.84 | 1.87 | 1.26 |
|  | 14. I am rarely bored on the courses. | 1.95 | 1.06 | 2.08 | 0.90 | 2.11 | 1.19 |
|  | 15. I have good friends in this school. | 3.42 | 0.84 | 3.36 | 0.86 | 3.28 | 0.98 |
|  | 19. My social life is good. | 2.98 | 0.99 | 3.16 | 0.92 | 2.79 | 1.12 |
|  | 28. I seldom (rarely) feel lonely. | 2.44 | 1.21 | 2.76 | 1.06 | 2.49 | 1.18 |
|  | 46. My accommodation is pleasant. | 3.29 | 1.02 | 3.39 | 0.96 | 3.38 | 0.87 |

M (SD): mean value (standard deviation)

Supplementary Table 1. Mean scores and standard deviations of the DREEM scales and subscales

| **Items** | **Factors** | | | | |
| --- | --- | --- | --- | --- | --- |
|  | **I** | **II** | **III** | **IV** | **V** |
| Factor 1: Students’ Perceptions of Learning | | | | | |
| 1 | 0.487 |  |  |  |  |
| 7 | 0.712 |  |  |  |  |
| 13 | 0.735 |  |  |  |  |
| 16 | 0.718 |  |  |  |  |
| 20 | 0.745 |  |  |  |  |
| 22 | 0.699 |  |  |  |  |
| 24 | 0.707 |  |  |  |  |
| 25 | 0.268 |  |  |  |  |
| 38 | 0.672 |  |  |  |  |
| 44 | 0.651 |  |  |  |  |
| 47 | 0.506 |  |  |  |  |
| 48 | 0.452 |  |  |  |  |
| Factor 2: Students’ Perceptions of Teachers | | | | | |
| 2 |  | 0.545 |  |  |  |
| 6 |  | 0.495 |  |  |  |
| 8 |  | 0.507 |  |  |  |
| 9 |  | 0.286 |  |  |  |
| 18 |  | 0.548 |  |  |  |
| 29 |  | 0.712 |  |  |  |
| 32 |  | 0.678 |  |  |  |
| 37 |  | 0.614 |  |  |  |
| 39 |  | 0.381 |  |  |  |
| 40 |  | 0.533 |  |  |  |
| 50 |  | 0.437 |  |  |  |
| Factor 3: Students’ Academic Self-Perceptions | | | | | |
| 5 |  |  | 0.302 |  |  |
| 10 |  |  | 0.384 |  |  |
| 21 |  |  | 0.727 |  |  |
| 26 |  |  | 0.475 |  |  |
| 31 |  |  | 0.484 |  |  |
| 41 |  |  | 0.688 |  |  |
| 45 |  |  | 0.584 |  |  |
| Factor 4: Students’ Perceptions of Atmosphere | | | | | |
| 11 |  |  |  | 0.483 |  |
| 12 |  |  |  | 0.410 |  |
| 17 |  |  |  | 0.083 |  |
| 23 |  |  |  | 0.607 |  |
| 30 |  |  |  | 0.655 |  |
| 33 |  |  |  | 0.663 |  |
| 34 |  |  |  | 0.659 |  |
| 35 |  |  |  | 0.460 |  |
| 36 |  |  |  | 0.507 |  |
| 42 |  |  |  | 0.687 |  |
| 43 |  |  |  | 0.748 |  |
| 49 |  |  |  | 0.607 |  |
| Factor 5: Students’ Social Self-Perceptions | | | | | |
| 3 |  |  |  |  | 0.522 |
| 4 |  |  |  |  | 0.484 |
| 14 |  |  |  |  | 0.460 |
| 15 |  |  |  |  | 0.555 |
| 19 |  |  |  |  | 0.605 |
| 28 |  |  |  |  | 0.528 |
| 46 |  |  |  |  | 0.330 |

Supplementary Table 2. Standardized factor loadings for the five-factor CFA model of the Hungarian DREEM (n=529)

| **Subscales** | **I** | **II** | **III** | **IV** | **V** |
| --- | --- | --- | --- | --- | --- |
| I. Perceptions of Learning | 1 |  |  |  |  |
| II. Perceptions of Teachers | 0.968 | 1 |  |  |  |
| III. Academic Self-Perceptions | 0.994 | 0.934 | 1 |  |  |
| IV. Perceptions of Atmosphere | 0.904 | 0.888 | 0.950 | 1 |  |
| V. Social Self-Perceptions | 0.833 | 0.767 | 0.868 | 0.927 | 1 |

Supplementary Table 3. Standardized factor correlations for the five-factor CFA model of the Hungarian DREEM (n=529)
